# Detection of Novel Biallelic Causative Variants in *COL7A1* Gene by Whole-Exome Sequencing, Resulting in Congenital Recessive Dystrophic Epidermolysis Bullosa in Three Unrelated Families

**DOI:** 10.3390/diagnostics12071525

**Published:** 2022-06-23

**Authors:** Fozia Fozia, Rubina Nazli, May Mohammed Alrashed, Hazem K. Ghneim, Zia Ul Haq, Musarrat Jabeen, Sher Alam Khan, Ijaz Ahmad, Mohammed Bourhia, Mourad A. M. Aboul-Soud

**Affiliations:** 1 Institute of Basic Medical Sciences (IBMS), Khyber Medical University (KMU), Peshawar 25100, Khyber Pakhtunkhwa, Pakistan; drfoziazeb@yahoo.com; 2 Department of Biotechnology and Genetic Engineering, Kohat University of Science & Technology (KUST), Kohat 26000, Khyber Pakhtunkhwa, Pakistan; sakmarwat79@gmail.com; 3 Chair of Medical and Molecular Genetics Research, Department of Clinical Laboratory Sciences, College of Applied Medical Sciences, King Saud University, P.O. Box 10219, Riyadh 11433, Saudi Arabia; malrashed@ksu.edu.sa (M.M.A.); hghneim@ksu.edu.sa (H.K.G.); 4 Institute of Public Health & Social Sciences (IPH&SS), Khyber Medical University (KMU), Peshawar 25100, Khyber Pakhtunkhwa, Pakistan; drzia@kmu.edu.pk; 5 Department of Gynaecology and Obstetrics, KMU Institute of Medical Sciences, Kohat 26000, Khyber Pakhtunkhwa, Pakistan; jabinmusarat@kmu.edu.pk; 6 Department of Chemistry, Kohat University of Science & Technology (KUST), Kohat 26000, Khyber Pakhtunkhwa, Pakistan; drijaz_chem@yahoo.com; 7 Laboratory of Chemistry, Biochemistry, Nutrition, and Environment, Faculty of Medicine and Pharmacy, University Hassan II, Casablanca 20000, Morocco; bourhiamohammed@gmail.com

**Keywords:** dystrophic epidermolysis bullosa, autosomal recessive, whole-exome sequencing, *COL7A1* gene, sequence variants

## Abstract

**Background:** Dystrophic Epidermolysis bullosa (DEB) is a rare, severe subtype of epidermolysis bullosa (EB), characterized by blisters and miliary rashes of the skin. Dystrophic EB (DEB) includes variants inherited both in an autosomal-dominant or autosomal-recessive manner. Recessive dystrophic EB (RDEB) is divided into many subtypes and prevails as a result of biallelic genetic mutations in *COL7A1* gene encoding type VII collagen, a major stabilizing molecule of the dermo-epidermal junction. The blister formation is mainly due to the variable structural and functional impairment of anchoring fibrils in VII collagen (COLVII), responsible for the adhesion of the epidermis to the dermis. **Method:** Three Pakistani families (A, B and C) affected with congenital dystrophic epidermolysis bullosa were recruited in the present study. The whole-exome sequencing (WES) approach was utilized for the detection of the pathogenic sequence variants in probands. The segregation of these variants in other participants was confirmed by Sanger sequencing. **Results:** This study identified a novel missense variant c.7034G>A, p. Gly2345Asp in exon 91, a novel Frameshift mutation c.385del (p. His129MetfsTer18) in a homozygous form in exon no 3, and a previously known nonsense variation (c.1573 C>T; p. Arg525Ter) in exon 12 of *COL7A1* gene in families A, B, and C, respectively, as causative mutations responsible for dystrophic epidermolysis bullosa in these families. **Conclusion:** Our study validates the involvement of the *COL7A1* gene in the etiology of dystrophic epidermolysis bullosa. It further expands the *COL7A1* gene mutation database and provides an additional scientific basis for diagnosis, genetic counseling, and prognosis purposes for EB patients.

## 1. Introduction

Recessive dystrophic epidermolysis bullosa (RDEB) is a very infrequent, aggressive subtype of dystrophic epidermolysis bullosa (DEB), a hereditary ailment starting right from birth in the form of trauma-induced blistering and scare formation of the skin. This disease prevails consequently due to biallelic genetic mutations in the *COL7A1* gene, which is responsible for collagen type VII (C7) production. Due to these mutations, C7 becomes absent or shows various anomalies in structure leading to disruptions in anchoring fibrils, which are the main securing bodies of the skin’s dermal and epidermal layers [1]. As a result of these mutations, the dermal layer of the affected individuals is always exposed to blister formations with minor trauma. RDEB is divided into numerous subtypes: severe form, an intermediate form and, other, rare, subtypes which include localized, pruriginosa and inversa [2]. Cutaneous manifestations of the RDEB include blister formation and development of wounds as a result of minor mechanical traumas, atrophic scarring and milia creation, nails dystrophy, and alopecia. Pruritus is also a frequent complaint. The individuals affected with severe and intermediate RDEB are more prone to develop squamous cell carcinoma (SCC) due to chronic wound formation and fibrosis which result in early death and reduced life expectancy in those patients [2,3]. Pseudosyndactyly, growth retardation, dental caries, anemia, esophageal strictures, malnourishment, and visual manifestations are some of the extracutaneous symptoms [1]. There are currently no authorized RDEB disease-modifying treatments. The disease’s therapy is confined to symptom management and secondary complications such wound care, trauma prevention, infection treatment, pain and itch management, strategically wrapping of the feet and hands to avoid pseudosyndactyly, and early diagnosis and treatment of SCC [1,4,5]. The disease’s gastrointestinal manifestations are handled with nutritional support, such as gastrostomy feeding, dilatation of an esophagus, and curing anemia [1,5]. Physiotherapy and rehabilitation, psychological and social provisions, and extra educational campaigns are all elements of disease management [1,4,6].

The most definitive tests for genetic analysis are a combination of gene-targeted testing (concurrent or, multigene panel, serial single-gene testing or single-gene testing), or more inclusive genomic testing (whole genome sequencing, chromosomal microarray analysis, or exome sequencing) based on the phenotype, but direct immunofluorescence (IF) and/or transmission electron microscopy (EM) may be useful, especially in the classification of its subforms [7].

In the present study, the WES method was applied for the mutational analysis of EB- affected patients in three families from three different provinces of Pakistan. A comprehensive clinical investigation followed by WES identified a novel biallelic missense mutation c.7034G>A, p. Gly2345Asp in exon 91, a novel frameshift mutation c.385del (p. His129MetfsTer18) in homozygous form in exon no 3 and a known homozygous nonsense mutation c.1573 C>T (p. R525*) in exon no 12 of the *COL7A1* gene.

## 2. Materials and Methods

### 2.1. Ethical Approval

This research project was conducted after taking approval from the Ethical Review Committee (ERC) of Institute of Basic Medical Sciences (IBMS), Khyber Medical University (KMU), Peshawar, Ethical code no Dir/KMU-EB/HM/000741/Dated 8/10/2020 and Ethical and Research Committee of Kohat University of Science and Technology (KUST), Kohat, Khyber Pakhtunkhwa Pakistan, Ref.No.VC-KUST/ethical committee/16-25/26.04.2016. and is in accordance with Helsinki declaration recommendations. All participants, including their parents or legal guardians, signed a written consent form.

### 2.2. Family Recruitment

**Figure 1:**
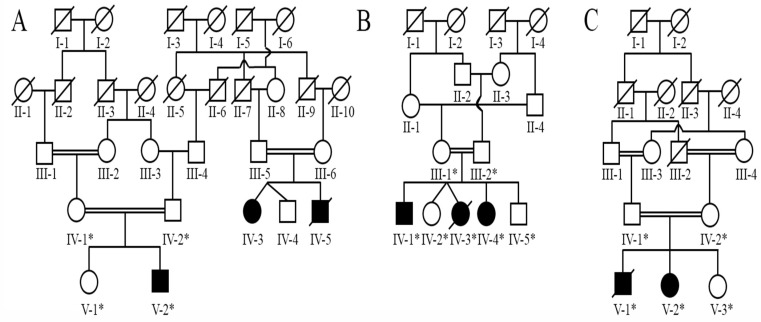
Pedigrees of Families (**A**–**C**) segregating Dystrophic epidermolysis bullosa in autosomal recessive manner. Double lines represent consanguineal union. Squares and circles indicate male and female members, respectively. Clear symbols indicate unaffected members, whereas filled symbols indicate affected members. An asterisk indicates that samples were accessible for the studies.

We recruited three unrelated families (A, B, and C) from three provinces of Pakistan (Figure 1). Pedigrees were constructed using information obtained from well-informed senior members of the individual lineages. In all three families, the pedigree revealed autosomal recessive inheritance.

### 2.3. Blood Sample Collection

Venous blood was drawn from 14 members of three families, including three people who were impacted (IV-3, IV-5, V-2), and two phenotypically normal individuals (IV-1, IV-2) in “Family A”, two affected (V-1, V-4) and three normal persons (IV-1, IV-2 and V-5) in “Family B”, and one patient (V-2) and three normal individuals (IV-1, IV-2, V-3) in “Family C”, in EDTA-coated tubes (BD Vacutainer K3, Franklin Lakes, NJ, USA). A professional dermatologist conducted a complete clinical assessment of the affected members of all the three families.

### 2.4. Genomic DNA Extraction

Extraction of human genomic DNA from the venous blood of all the participating individuals was done with the help of genomic DNA extraction kit (QIAGEN, Germantown, MD, USA) ensuing the manufacturer’s manual guiding principle.

### 2.5. Whole-Exome Sequencing and Segregation of Rare Variants through Sanger Sequencing

DNA samples of an affected proband from all three families were submitted to 3Billion for whole-exome sequencing (WES). The exon domains of all human genes (22,000) were retrieved by the xGen Exome Research Panel v2 (Integrated DNA Technologies, Coralville, IA, USA). The depicted genomic regions were sequenced by Novaseq 6000 (Illumina, San Diego, CA, USA). 3Billion used open-source bioinformatics tools and in-house computer software to analyze raw (genome sequencing) data, including alignment (to the GRCh37/hg19 human reference genome), variation calling, and annotation. The automatic variant interpretation software, EVIDENCE, was developed in-house to prioritize variants based on the ACMG guidelines (Genet. Med. 2015;17:405–424) and the phenotype of each affected individual. This system has three major phases: filtration of variants, classification and the patient’s phenotype similarity scoring (Clin. Genet. 2020; 98:562–570). Initially, allelic frequency was estimated by utilizing databases of 3Billion and gnomAD (http://gnomad.broadinstitute.org/, accessed on 20 June 2021) as reference databases for the population genome. Filtration of common mutations (having MAF of >5%) were performed in accordance with the ACMG guideline BAI (Genet. Med. 2015; 17:405–424). Secondly, a number of databases, including ClinVar (https://www.ncbi.nlm.nih.gov/clinvar/, accessed on 20 June 2021) and UniProt (https://www.uniprot.org/, accessed on 20 June 2021), were utilized to extricate the pathogenicity of each identified variant with its related diseases in accordance with the ACMG guideline reference recommendations (Genet. Med. 2015;17:405–424). Third, the observed phenotypes of the patients were converted to their comparable standardized human phenotype ontology terms (https://hpo.jax.org/, accessed on 20 June 2021) and retrieved to calculate the similarities (Am. J. Hum. Genet. 2016;98:490–499 and Am. J. Hum. Genet. 2009;85:457–464) with each of ~7000 rare inherited disorders (https://omim.org/ and https://www.orpha.net/consor/cgi-bin/index.php, accessed on 20 June 2021). The similarity scoring patient’s phenotype and the symptoms associated with that disease caused by prioritized variants based upon the ACMG guidelines varied from 0 to 10.

Several databases, including the 1000 genome project (https://www.internationalgenome.org/, accessed on 20 June 2021), dbSNP (https://www.ncbi.nlm.nih.gov/snp/, accessed on 20 June 2021), EVS (https://evs.gs.washington.edu/EVS/, accessed on 20 June 2021), and gnomAD (https://gnomad.broadinstitute.org/, accessed on 20 June 2021), were utilized for filtration and validation of identified genetic variants obtained from WES analysis. The University of California Santa Cruz (UCSC) genome database browser (http://genome.ucsc.edu/cgibin/hgGateway, accessed on 20 June 2021) was used to obtain the reference sequence (Table 1). Primer3Plus (http://www.bioinformatics.nl/cgibin/primer3plus/primer3plus.cgi/, accessed on 20 June 2021) was used for designing the primers for PCR amplification (Supplementary Materials Table S1). Subsequent sequencing of genetic DNA was accomplished by the ABI3730 genetic analyzer with BigDye chemistry v3.1. A sequence alignment program, BioEdit version 6.0.7 (http://www.mbio.ncsu.edu/BioEdit/bioedit.htm, accessed on 20 June 2021), was used to align the obtained sequences against the reference sequence.

#### Pathogenicity Prediction and Protein Sequence Alignment

For performing Insilco evaluation, different pathogenicity detection tools were used, including MutationTaster (http://www.mutationtaster.org/, accessed on 20 June 2021), PROVEN (http://provean.jcvi.org/seq_submit.php), PolyPhen2 (http://genetics.bwh.harvard.edu/pph2/, accessed on 20 June 2021), FATHMM (http://fathmm.biocompute.org.uk/inherited.html, accessed on 20 June 2021). PROVEAN (SIFT (https://sift.bii.astar.edu.sg/, accessed on 20 June 2021), VarSome (https://varsome.com/, accessed on 20 June 2021), and MutPred2 (http://mutpred.mutdb.org/, accessed on 20 June 2021). A sequence alignment programme, BioEdit version 6.0.7 (http://www.mbio.ncsu.edu/BioEdit/bioedit.htm, accessed on 20 June 2021), was used to align the sequences against the reference sequence (Table 2).

## 3. Results

### 3.1. Clinical Phenotypes

**Figure 2:**
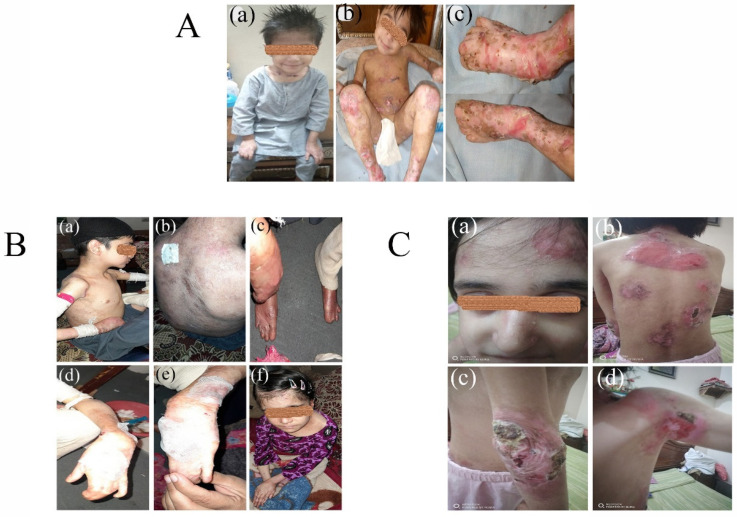
(**A**) Clinical phenotypes of epidermolysis Bullosa in “**Family A**”. Patient V-2 shows classical representation of Recessive Dystrophic EB, generalized severe type with abnormal blistering of the skin, erosions and atypical scarring with erythematous hyperkeratotic papule and keloids affecting his trunk, arms, hands, feet, elbows, knees and genitalia (**Aa**–**c**), nail dystroph, anonychia (**a**), fusion-induced mitten abnormalities of the hands and toes, joint contractures, pseudo yndactyly and growth delay (**Aa**–**c**). (**B**) Clinical phenotypes of epidermolysis Bullosa in “**Family B**”. The two affected Patients V-1 and V-4 from “Family B” present cardinal signs of recessive dystrophic EB, generalized severe type with abnormal generalized blistering of the skin and extensive scarring of the whole body (**Ba**–**f**), with thin and spares scalp hairs (**Bf**), nail dystrophy and onycholysis (**Bc**,**d**), atrophic scarring, malia (**Ba**,**b**,**c**,**f**), pseudo syndactyly and flexion contracture of joints (**Ba**,**c**,**d**,**e**). (**C**) Clinical phenotypes of epidermolysis Bullosa in “**Family C**”. A 13-year-old girl (V-2) in family C shows typical features of DEB, including abnormal blistering of the skin, erosions and atypical scarring with erythematous hyperkeratotic papule and keloids of skin affecting trunk and extremities (**Cb**,**c**,**d**). She has nail dystrophy, joint contractures, severe blistering of oral mucosa and esophageal strictures causing swallowing and feeding difficulties. She also faces severe anemia, growth delay, frequent corneal and conjunctival erosions, symblepharon and, exposure keratitis (**Ca**).

A 4-year-old boy (V-2) from Family A had a history of abnormal blister formation on the skin, erosions, and atypical scarring with erythematous hyperkeratotic papule, and keloids affecting his trunk, arms, hands, feet, elbows, knees and genitalia. He had nail dystrophy, small nails and anonychia (Figure 2(Aa,c)). His mother gives a history of fragile erythematous blistering skin since birth, which does not improve with time. He also has fusion-induced mitten abnormalities of the hands and toes, as well as joint contractures, palmoplantar keratoderma, oral mucosal blisters with feeding difficulties, hypoplasia of dental enamel, carious teeth, pseudo syndactyly, and growth delay. There is the history of frequent inflammation of the eyes, skin, gastrointestinal and respiratory systems.

The proband (V-1) in Family B demonstrates abnormal generalized blistering of the skin and extensive scarring of the whole body. Opening of the mouth is restricted along with ankyloglossia and feeding difficulties. The limbs show nail dystrophy in form of onychomadesis and onycholysis, atrophic scarring, malia, pseudo syndactyly and restriction of movement due to flexion contracture of joints. Other findings include corneal and conjunctival blistering, anemia, constipation, muscle pains, growth delay, and chronic infections of the respiratory, gastrointestinal, and urogenital systems (Figure 2(Ba–f)).

A 13-year-old girl (V-2) in Family C, shows typical features of DEB, including abnormal blistering of the skin, erosions, and atypical scarring with erythematous hyperkeratotic papule and keloids of the skin affecting the trunk and extremities, showing no improvement with advancing age. She has nail dystrophy, along with mitten deformities of phalanges, joint contractures, severe blistering of the oral mucosa and esophageal strictures, causing swallowing and feeding difficulties. She also faces severe anemia, growth delay, frequent corneal and conjunctival erosions, symblepharon, anterior blepharitis, exposure keratitis, and conjunctival complications along with other organ system infections (Figure 2(Ca–d)).

#### Screening of Pathogenic Sequence Variants

Analysis of the WES data results in identification of two, homozygous novel mutations (c.7034G>A, p. Gly2345Asp and c.385del (p. His129MetfsTer18) and one known homozygous variant (c.1573 C>T (p. R525*) in the *COL7A1* gene in diseased individuals of three families (A, B, and C), causing RDEB. The obligate carriers were heterozygous for the detected pathogenic variations, whereas the affected individuals were homozygous. The 100 ethnically matched healthy controls were evaluated to rule out the chance of polymorphism among the identified mutations., removing the possibility of nonpathogenic polymorphisms for those unique mutations.

**Figure 3:**
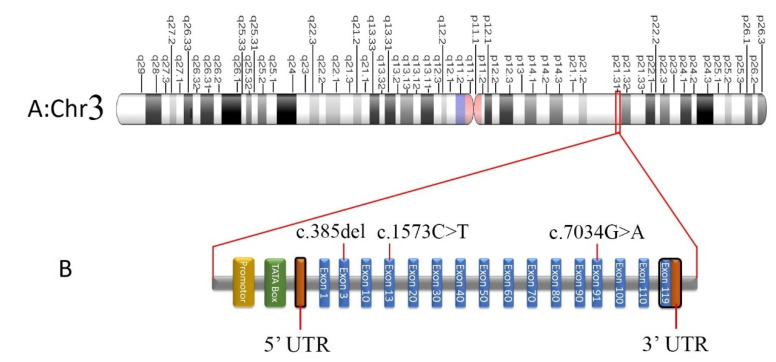

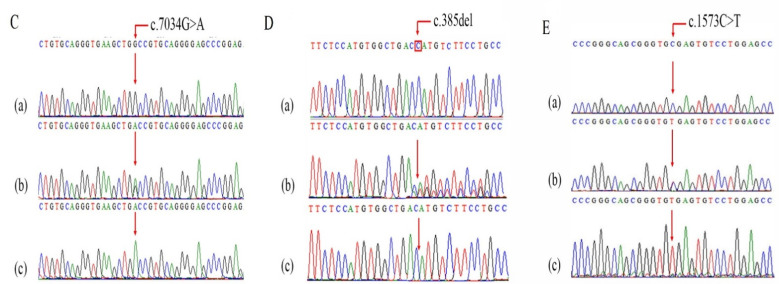
(**A**) Hypothetical structure of human chromosome 3 encoding *COL7A1* gene on chromosome q21, shown by red box. (**B**) Hypothetical presentation of *COL7A1* gene, consisting of 118 exons. (**C**) Sequencing analysis of exon 91 of *COL7A1* gene in family A, showing a novel missense variant (c.7034G>A, p.Gly2345Asp) in the affected sibling (V-2) born to a consanguineous union: (**Ca**) affected (V-2), (**Cb**) obligate carriers or parent(IV-1), (**Cc**) wild type or unrelated healthy control. (**D**) Sequencing analysis of exon 3 of *COL7A1* gene in family B, showing a frameshift mutation c.385del (p.His129MetfsTer18) in homozygous form in the affected sibling (V-1) born to a consanguineous union: (**Da**) wild type or unrelated healthy control, (**Db**) obligate carriers or parent(IV-1), (**Dc**) affected (V-1). (**E**) Sequencing analysis of exon 12 of *COL7A1* gene in family C, showing a nonsense variation (c.1573 C>T; p. Arg525Ter) in the affected sibling (V-2) born to a consanguineous union: (**Ea**) affected (V-2), (**Eb**) obligate carriers or parent (IV-1), (**Ec**) wild type or unrelated healthy control. The solid red lines represent positions of the mutations.

In “Family A”, the identified a novel missense variant c.7034G>A, p. Gly2345Asp lies in exon 91 of *COL7A1*. Sanger sequencing confirmed identified variant (c.7034G>A) in the proband (V-2). Afterward, the variant was screened in parents (IV-1 and IV-2) and found to cosegregate within the family with the phenotype of disease. Patient (V:2) was carrying the mutation in homozygous state (Figure 3(Ca)), whereas his parents (IV-1 and IV-2) were heterozygous for the mutant allele (Figure 3(Cb)). The homozygous mutation c.7034G>A in *COL7A1* changes an amino acid Gly to Asp at 2345codon in 91number exon. SIFT, Polyphen2 and Fathmm predicted the variant c.7034G>A to be damaging, likely pathogenic by Varsome and MutPred, disease causing by Mutation Taster and deleterious by PROVEAN. It was also shown to have decreased stability by MUPRO (Table 2).

In Family B, WES revealed a frameshift mutation c.385del (p. His129MetfsTer18) in homozygous form in the 3rd exon of *COL7A1*. The selected c.385del mutation of *COL7A1* was confirmed in homozygous state in patient IV-4 through Sanger sequencing (Figure 3(Da)). The variant was co-segregated within the family with the disease phenotype. Consistent with the autosomal recessive mode of inheritance, the parents (II:1 and II:2) and the phenotypically normal individual (III:2) were in a heterozygous state for the c.385del mutation (Figure 3(Db,c)). The homozygous variant c.385del on *COL7A1* deletes nucleotides ‘C’ from exon 3, which creates frameshift change in the protein coding sequence. The deletion is expected to cause protein truncation. This is likely to result in normal protein function loss, due to nonsense-mediated mRNA decaying. These two variants in *COL7A1* are predicted to be novel, as they have never been shown in gnomAD [8] HGMD [9], Clinvar [10] or the Pakistan Genetic Mutation Database (PGMD) [11].

**Figure 4:**
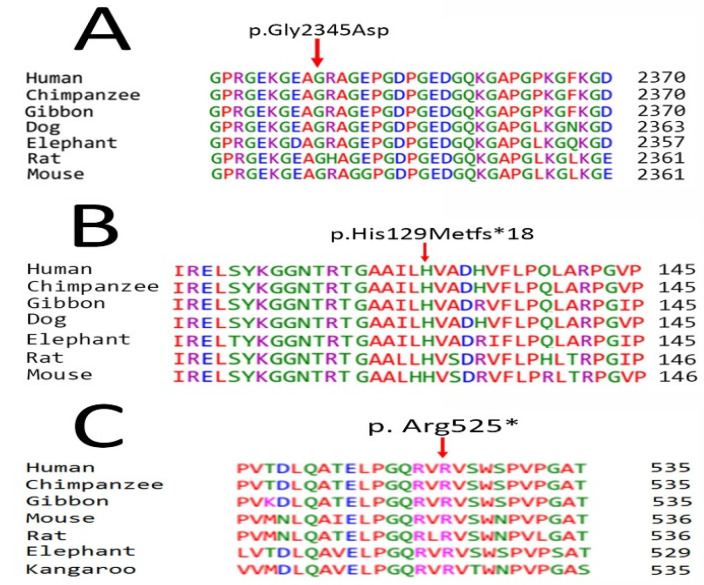
Multiple sequence alignment of Human Gly2345 in Family **A**, Human His129 in Family **B** and Human Arg525 in Family **C** with their orthologs. Red arrows showing their evolutionary conservation through different species.

A nonsense variation (c.1573 C>T; p. Arg525Ter) in the *COL7A1* 12th exon was discovered in Family C, and its segregation was verified by Sanger sequencing in other participants (Figure 3E(a–c)). In the *COL7A1* gene, this genetic alteration causes a premature translational stop signal (p. Arg525*). It is likely to result in a protein product that is missing or disrupted. In population databases, this variant can be found (rs368007918, ExAC 0.006%). Individuals with autosomal recessive epidermolysis bullosa have been found to carry this variation (PMID: 10504458, 21448560). This variation has a record in ClinVar (Variation ID: 279785). This variant may produce or brace a splicing site, according to algorithms built to anticipate the effect of sequence alterations on RNA splicing, although not verified yet. The *COL7A1* function loss mutation is considered to be disease causing (PMID: 16971478) (Table 2). This variation has been labeled as pathogenic as a result of these factors. Figure 4A–C display multiple sequence alignment of Human Gly2345, His129, and Arg525 with their orthologs in different species.

**Table 1:** Information on *COL7A1* variants.

Gene	Variant	Chr.	Chr. Position	Variant Type	Cyto Band	GenBank	Ref. Seq.	Alt. Seq.	RS ID/dbSNP	UniProt ID	MAF gnomAD	MAF gnomAD South Asian
*COL7A1*	c.7034G>A; p.Gly2345Asp	3	**48609468**	SNV	**3p21.31**	NM_000094.4	C	T	NR	Q02388	NR	NR
*COL7A1*	c.385delG; p.His129MetfsTer18	3	**48631011**	Deletion	**3p21.31**	NM_000094.4	G	-	NR	Q02388	NR	NR
*COL7A1*	c.1573C>T p.R525X	3	**48628960**	SNV	**3p21.31**	NM_000094.4	G	A	**rs368007918**	Q02388	**0.00000796**	**0.0000327**

**Table 2:** Pathogenicity detection of *COL7A1* variants.

Gene	Variant	MT	PolyPhen2	MUPRO	Fathmm	VarSome	PROVEAN	MutPred	SIFT
*COL7A1*	c.7034G>A; p.Gly2345Asp	Disease causing	**Possibly** **Damaging**	Decrease stability	Damaging	Likely pathogenic	Deleterious	Pathogenic	Damaging
*COL7A1*	c.385delG; p.His129MetfsTer18	Disease causing	NA	NA	NA	Pathogenic	NA	NA	NA
*COL7A1*	c.1573C>T p.R525X	Disease causing	NA	NA	Neutral	Pathogenic	NA	NA	NA

## 4. Discussion

Patients suffering from epidermolysis bullosa (EB) face lifelong discomfort and treatment dilemmas, particularly in Pakistan, where health care facilities have poor availability. Rare genetic diseases are more frequent in this population because of a deficit in genetic testing facilities and poor information for diseased families.

All types of DEB are caused by genetic defects of the *COL7A1* gene, which translates type VII collagen [12]. Type VII collagen is coded by *COL7A1,* spanning 118 exons on 3p21.10 chromosome. The mRNA transcript of ~8.9 kb produce procollagen α1 (VII) chain consisting of 2944 amino acids [12]. The three main structures of each polypeptide chain are: a collagenous triple helix (coded by 29 to 112 exons), an amino terminus NC-1 (coded by exons 2 to 28 exons) and a carboxyl terminus NC-2 (coded by 113 to 118 exons) [13]. The formation of anchoring fibrils takes place by deposition of collagen VII underneath the basement membrane of the epidermis of the skin where it is responsible for the connection of dermal ECM to the basement membrane. Deficient collagen VII causes persistent fragility of the skin, ongoing fibrosis of multiple organ systems, and scarring [14].

In severe generalized RDEB, homozygous or compound heterozygous mutations in the *COL7A1* gene produce premature termination codons, leading to absence of anchoring fibril production due to degradation of abnormally truncated polypeptides [15]. Fritsch [16] created a transgenic mouse model with contingent inactivation of *COl7A1* expression, resulting in a *COl7A1* hypomorphic animal expressing about 10% of normal *COl7A1* and clinical symptoms that closely resemble human recessive abnormalities, such as nail dystrophy, skin fragility, and growth failure. Clinically, RDEB-GS patients present with the involvement of the integumentary system, including the mucous membranes (Figure 2), esophageal strictures necessitating gastrostomy tube placement, mitten deformities of the hands, toes and fingers, loss of nail plates, joint contractures, and eye inflammation with visual impairment [17]. Additionally, these excessive heavily fibrotic lesions favor the progressive formation of aggressive cutaneous squamous cell carcinoma (SCC) at an early age, the peritumoral infiltration of immune cells causes immunosuppression and metastasis development [18,19], decreasing their life expectancy to only their fourth decade [20]. Many of the same clinical characteristics are present in RDEB-GO individuals; however, they are milder. The non-severe variants have a little better prognosis, with a median survival age of 55 to 65 years, basically because of immunosuppression and immunosurveillance which are responsible for onset and progression of malignant lesions at the molecular level [21].

Although a cure for EB is still a long way off, recent findings in clinical trials based on humans and animal models have encouraged affected individuals, clinicians, and genetic investigators to believe that modification of the disease factors and enhancement of quality of life are achievable motives [4,14]. In this regard, many scientists are determining the efficacy of certain drugs, ointments, and gene therapy in treating Epidermolysis bullosa, especially in *COL7A1* mutation-affected patients. Webber et al., 2017 [20] used a (CRISPR/Cas9) method with microinjection into NOD/SCID IL2rcnull (NSG) embryos to swiftly construct an immunodeficient Col7a1/animal model of RDEB for experimental purposes. They also recognized the ability of a newly discovered skin-resident immunomodulatory dermal mesenchymal stem cell, ABCB5, to minimize disease and significantly increase the life expectancy of these (RDEB NSG) mice by reducing skin inflammatory myeloid-derived penetration. Double-strand breaks are induced via Nuclease-based genome editing at certain genomic sites (Porteus, 2019). Several approaches for modifying the genomes of fibroblasts and keratinocytes for therapeutic purposes have been successful [22,23]. Changes in mutant allele reading frames for knockout [24,25,26] or coding sequence restoration [27,28,29] have been shown to be very effective. Dual RNP-mediated genome editing is efficient and homogenous, eliminating the need for plasmid-based delivery and selecting properly altered single-cell clones [30]. Moreover, recent advancements have uncovered more comprehensive, expectable consequences ensuing single targeting through CRISPR/Cas9 at various loci [31,32]. Kocher used a CRISPR/Cas9 nuclease as a ribonucleoprotein to induce a consistent, predicted single adenine sense-strand insertion at the target position in primary wild-type and recessive DEB keratinocytes [33]. This shows that *COL7A1* editing based on precise end-joining-mediated DNA repair is an efficient strategy for reversing the characteristics of DEB associated with the disease, regardless of the inheritance pattern of the disease.

Similarly, Mayr et al., 2022 showed for the first time the potential of endogenous 50 trans-splicing as a useful tool for beneficially modulating the RDEB-phenotype by correcting pathogenic mutations within the COL7A1 gene, thereby addressing an essential need of this patient population [34].

In this study, Family A was diagnosed as being affected by epidermolysis bullosa Recessive Dystrophic EB, generalized severe type, which is a rare sub-type of DEB, associated with mutations of the *COL7A1* gene. Clinically, it is characterized by sublamina densa blistering that leads to erosions and poor wound healing, persistent ulcers, and significant scarring, notably on the hands and feet, which are exposed to frequent trauma [35]. The proband, a 4-year-old boy (V-2) in Family A, had a history of abnormal blister formation on the skin, erosions and atypical scarring with erythematous hyperkeratotic papules, and keloids affecting his trunk (Figure 2(Aa–c)), arms, hands, feet, elbows, knees, and genitalia. He also had nail dystrophy, mitten deformities, pseudo syndactyly and dysphagia, as described in previous studies [17,36]. WES revealed a novel missense mutation variant c.7034G>A, p. Gly2345Asp in exon 91 of *COL7A1* (Table 1). This variant c. 7034G>A was predicted to be disease producing by Mutation Taster, damaging by Polyphen-2, SIFT and Fathmm, and pathogenic by VarSome and MudPred, and was shown to have decreased stability by MUPRO (Table 2).

In Family B, homozygous novel frameshift sequence variant c.385del (p. His129MetfsTer18) in the 3rd exon of the *COL7A1* gene was identified by WES, which results in generalized severe RDEB in the affected members of the family (Table 1). The proband IV-4 demonstrated abnormal generalized blistering of the skin and extensive scarring of the whole body. Involvement of the mucosal membranes has been documented in some patients, causing difficulty with oral feeding [36], as observed in our patient. Opening of the mouth was restricted, along with ankyloglossia and feeding difficulties. The limbs show onycholysis, poly syndactyly, and restriction of movement due to flexion contracture of joints. The mutation c.385del was predicted to be disease producing by MutationTaster and pathogenic by VarSome (Table 2).

In Family C, a 13-year-old girl (IV-2) presented with typical features of DEB, including abnormal blistering of the skin, erosions and atypical scarring with erythematous hyperkeratotic papules and keloids of skin affecting the trunk and extremities, showing no improvement with advancing age. Other symptoms included mitten deformities of phalanges, joint contractures, severe blistering of oral mucosa and esophageal strictures, feeding difficulties, severe anemia, growth delay, frequent corneal and conjunctival erosions, symblepharon, anterior blepharitis, exposure keratitis and conjunctival complications, as reported in other studies [37,38,39]. WES detected a nonsense sequence variant (c.1573 C>T; p. Arg525Ter) in *COL7A1* at the 12th exon, cosegregating with the diseased members of the family (Table 1). This genetic mutation in the *COL7A1* gene causes a premature translation stop signal (p. Arg525*) and is classed as pathogenic (Table 2).

Commonly practicing consanguineal marriages has turned out to be a distressing matter in an ethnically rich, religious country like Pakistan, further increasing the risk of many monogenic disorders (autosomal recessive). Under such circumstances, Next-Generation Sequencing technologies (WES and Whole-Genome Sequencing, WGS) are a preferred state-of-the-art molecular diagnostic tool for quick and efficient diagnosis of single-gene disorders. These novel mutations identified in the *COL7A1* gene causing RDEB in three different families will further elaborate the phenotypic and mutational spectrum of RDEB, and will also aid in the development of clinical and gene-based therapeutic strategies and targeted genome-editing techniques which, along with the procedures for inducing pluripotent stem cells, are predicted to permit and expand the usage of patient-specific autologous stem cell(renewable) therapies [40,41], especially in patients affected by *COL7A1* mutations, especially in Pakistan, where *COL7A1* mutations prevail in increased numbers, as shown in this article. In this sense, detecting and analyzing individual genetic mutation is a critical first step in devising suitable personalized treatment strategies, as well as the identification of individuals who could gain an advantage from particular innovative genomic therapies. The present study adds to the knowledge of clinical heterogeneity associated with *COL17A1* variants, and it will also help in genetic counseling and accurate prenatal diagnosis for parents of at-risk individuals in order to facilitate decision making regarding the continuation of upcoming pregnancies, especially in a region like Pakistan, where the custom of consanguineous marriage prevails at a much higher rate.

## Data Availability

The data presented in this study are available on request from the corresponding author.

## Supplementary Materials

The following are available online at https://www.mdpi.com/article/10.3390/diagnostics12071525/s1, Table S1: Primers used for the amplification of the regions of interest.
