# Supplementary material for: Detection of Novel Biallelic Causative Variants in COL7A1 Gene by Whole-Exome Sequencing, Resulting in Congenital Recessive Dystrophic Epidermolysis Bullosa in Three Unrelated Families
Source: Diagnostics (Basel). 2022 Jun 23;12(7):1525. doi: 10.3390/diagnostics12071525 (PMC9316163; doi:10.3390/diagnostics12071525)
Supplement: Supplementary file 1 [file diagnostics-12-01525-s001.zip › diagnostics-1747314-supplementary.pdf]

Table S1. Primers used for the amplification of the regions of interest.

Gene name	EXON/ INTRON number	primer sequence (5'-3')	Tm(c)	product size	Length	No of hits
COL7A1	91	LEFT PRIMER 5'- CTGTCTCTCCAGTGGCCTA -3'<br>RIGHT PRIMER 5'- CCCAGATGTGGTGAGAAAC -3'	56.40<br>56.32	583bp	19<br>19	1<br>2
COL7A1	3	LEFT PRIMER 5'- CCTGATACCCGTAACCCTCA -3'<br>RIGHT PRIMER 5'-CACTCCTGCTCGGTCCTTAC-3'	59.81<br>59.87	457bp	20<br>20	1<br>1
COL7A1	12	LEFT PRIMER 5'- GCTGGTCTGACCCTGTTATC -3'<br>RIGHT PRIMER 5'- AGACACACCCTGTTGACAGTT-3'	57.16<br>57.10	562bp	20<br>21	2<br>1
